# Incorporation of the Endoplasmic Reticulum Stress-Induced Spliced Form of XBP1 mRNA in the Exosomes

**DOI:** 10.3389/fphys.2018.01357

**Published:** 2018-09-26

**Authors:** Toru Hosoi, Mieko Nakashima, Koichiro Ozawa

**Affiliations:** Department of Pharmacotherapy, Graduate School of Biomedical and Health Sciences, Hiroshima University, Hiroshima, Japan

**Keywords:** exosome, unfolded protein response, endoplasmic reticulum stress, IRE1α, XBP1

## Abstract

It is known that endoplasmic reticulum (ER) and nucleus communicate with each other to cope with ER stress. However, the mechanisms through which extracellular transmission of ER stress occurs remain unexplored. When the ER stress-induced unfolded protein response (UPR) is activated, the X-box binding protein 1 (XBP1) mRNA is spliced by inositol-requiring enzyme-1α (IRE1α) to produce the spliced form of XBP1 (sXBP1). In the present study, we found that sXBP1 mRNA in the cell may be incorporated into the exosomes and was released extracellularly. We found that the ratio of the mRNA levels of sXBP1 to unspliced XBP1 (uXBP1) in the exosome was higher than that of cells in MIN6 mouse pancreatic β cells. A similar effect was observed when XBP1 splicing was induced by overexpressing IRE1α in HEK293T cells. These results suggest that the incorporation of sXBP1 into the exosomes is a novel mechanism of UPR transmitted to extracellularly, which would be triggered when cells are exposed to stress.

## Introduction

Cells encounter various environmental stress stimuli, which perturb the functioning of endoplasmic reticulum (ER). Such stimuli cause accumulation of unfolded proteins in the ER, which in turn induces the cells to activate the unfolded protein response (UPR) to cope with such a stress (ER stress) (Walter and Ron, 2011). To date, three major types of UPR stress transducer proteins have been identified: inositol-requiring enzyme-1 (IRE1), double stranded RNA-activated protein kinase (PKR)-like ER kinase (PERK), and activating transcription factor 6 (ATF6) (Ron and Walter, 2007). Activation of these stress transducer proteins conveys the stress signals to the nucleus. Activation of IRE1 triggers X-box binding protein 1 (XBP1) mRNA splicing, and the spliced form (sXBP1) enters the nucleus and acts as a transcription factor (Yoshida et al., 2001; Calfon et al., 2002).

Mounting evidence has shown that the ER and nucleus communicate with each other to cope with ER stress (Hetz, 2012). However, as far as we know, there is no previous evidence showing the extracellular transmission of ER stress through exosome. The exosomes are nanoscale vesicles (50–150 nm in diameter) that are secreted extracellularly. Increasing evidence suggests that the secreted exosomes play a role in cell–cell communication (Valadi et al., 2007; Schorey et al., 2015). In the present study, we hypothesized that the ER stress signals may be transduced and packed into the exosomes.

## Materials and Methods

### Materials and Reagents

Tunicamycin was obtained from Wako Pure Chemical Industries, Ltd. (Japan). ExoQuick-TC was obtained from System Biosciences (CA). Exosome-depleted FCS was obtained from System Biosciences (CA) or Life Technologies (CA). We wear gloves and lab coat and used hoods for the preparation of biohazard elements.

### Cell Culture

HEK293T cell line was cultured in DMEM with 10% fetal calf serum (FCS), 100 units/ml of penicillin G, 100 μg/mL of streptomycin, and 0.25 μg/mL of amphotericin B (Nacalai Tesque Inc., Japan). The MIN6 cell line was cultured in DMEM with 50 μM β-mercaptoethanol, 10% FCS, 100 units/mL of penicillin G, 100 μg/mL of streptomycin, and 0.25 μg/mL of amphotericin B (Nacalai Tesque Inc.). The cells were cultured at 37°C with 5% CO_2_.

### Transfection

On the starting day (day 0), the HEK293T cells were plated onto 100-mm dishes. On day 1, the cultured cells were replenished with fresh medium (DMEM, 10% FCS) not containing antibiotics. Three hours later, the cells were transfected with IRE1α (FLAG-tagged human IRE1α) plasmid by using the calcium phosphate co-precipitation method. Sixteen hours after the transfection, the cells were replenished with fresh medium (DMEM), which included exosome-depleted FCS (5%) and antibiotics.

### RT-PCR

Total RNA was isolated and RT-PCR was performed as previously described, albeit with a few modifications (Hosoi et al., 2016). For PCR amplification, we used the Expand High Fidelity kit (Roche AG) and KAPA Taq Extra PCR kit (KAPA Biosystems). The following primers were used: mXBP1 upstream, 5′-CCT TGT GGT TGA GAA CCA GG-3′; mXBP1 downstream, 5′-CTA GAG GCT TGG TGT ATA C-3′; hXBP1 upstream, 5′-CCT TGT AGT TGA GAA CCA GG-3′; hXBP1 downstream, 5′-GGG GCT TGG TAT ATA TGT GG-3′. The PCR products were resolved by electrophoresis with an 8% polyacrylamide gel and were stained with ethidium bromide.

### Western Blotting

Western blotting was performed as described previously (Hosoi et al., 2014) albeit with some modifications. Briefly, the cells were washed and lysed for 20 min with RIPA buffer containing 25 mM Tris HCl (pH 7.6), 150 mM NaCl, 1% NP-40, 0.1% SDS, 1% sodium deoxycholate, 1 mM Na_3_VO_4_, 10 mM NaF, 10 μg/mL aprotinin, 10 μg/mL leupeptin, and 1 mM phenylmethylsulfonyl fluoride (PMSF). The lysates were incubated on ice for 20 min. Then, the samples were sonicated for 4 min and suspended for 30 min at 4°C. After additional sonication for 4 min, the samples were centrifuged at 14,000 rpm for 5 min at 4°C, and the supernatants were collected. The samples were then boiled in SDS sample buffer for 3 min, fractionated with SDS-PAGE, and transferred to nitrocellulose membranes. These membranes were then incubated with anti-CD63 (Novus; 1:1000), anti-calnexin (Enzo; 1:1000), anti-CD81 (Cell signaling; 1:1000), and anti-IRE1α (Cell signaling; 1:1000) antibodies, followed by an anti-horseradish peroxidase-linked antibody. Peroxidase binding was detected using chemiluminescence with an enhanced chemiluminescence system.

### Collection of Exosomes

Exosomes were collected from the cell culture medium 24 h after replacement with fresh medium containing exosome depleted FCS. ExoQuick-TC was used to collect the exosomes from the culture supernatant by following the manufacturer’s instructions. The quality of the exosomes was checked by Western blotting with anti-CD63, anti-calnexin, and anti-CD81 antibodies.

### Electron Microscope Analysis

Exosomes obtained were analyzed by scanning electron microscopy (SEM). The isolated samples were loaded on electron microscopy grid and then fixed with 2% glutaraldehyde and 2% paraformaldehyde for 1.5 h at room temperature. After washing with PBS, the samples were stained with 2% phosphotungstic acid solution (pH7) for 20 min at room temperature. Then, the samples were dehydrated with ethanol (50, 70, 80, 90, 95, 100%), incubated with tert-butyl alcohol, and dried using freeze dryer. After the dehydration, samples were coated with osmium tetroxide and analyzed with Field Emission Scanning Electron Microscope (JSM-7800F, JEOL Ltd.).

### Statistics

**FIGURE 1:**
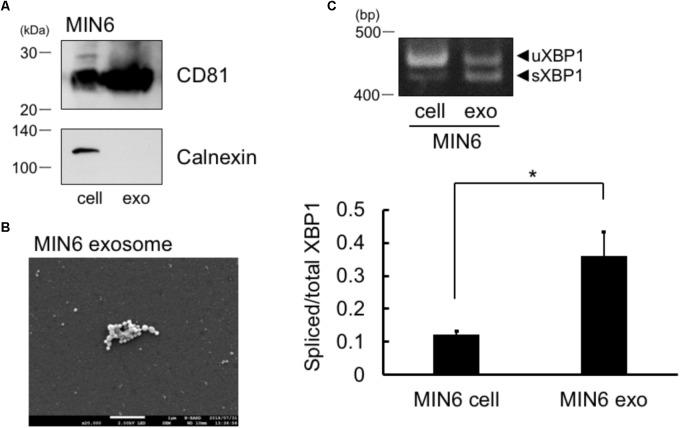
Spliced form of XBP1 mRNA was incorporated into the exosomes of pancreatic β cells. **(A)** Exosomes in the MIN6 cell culture supernatant expressed CD81 but not calnexin. MIN6 pancreatic β cells were cultured for 24 h in DMEM containing exosome-depleted FCS. Then, the exosomes were extracted from the supernatant, and CD81 and calnexin levels were measured by Western blotting. Typical data of 3 independent experiments was shown. Other 2 data were shown in the **Supplementary Figure 1A**. **(B)** Electron microscope analysis of the exosomes obtained from MIN6 pancreatic β cells. Scale bar: 1 μM. **(C)** MIN6 pancreatic β cells were cultured for 24 h in DMEM containing exosome-depleted-FCS. Then, the exosomes were extracted from the supernatant. The mRNA levels of total and spliced forms of XBP1 (sXBP1) were analyzed in cells (left band of the panel) and exosomes (right band of the panel) by RT-PCR, with specific primers for XBP1. Typical data of 4 independent experiments was shown. Other 3 data were shown in the **Supplementary Figure 1B**. ^∗^*P* < 0.05 by Student’s *t*-test

**FIGURE 2:**
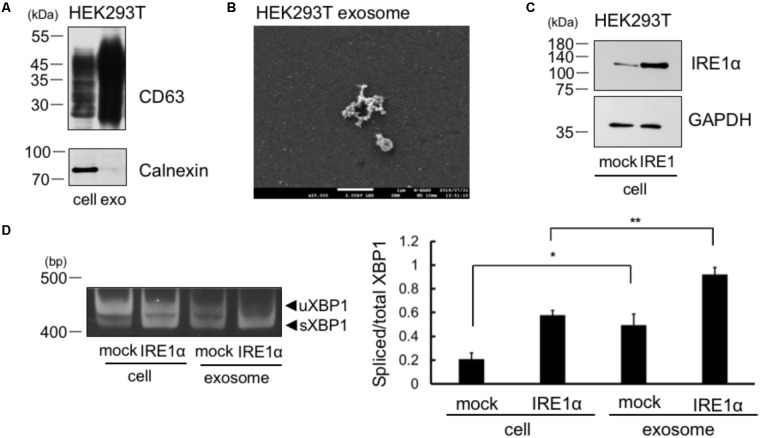
Spliced form of XBP1 mRNA was incorporated into the exosomes of HEK293T cells, which overexpress IRE1α. **(A)** HEK293T cells were cultured for 24 h in DMEM containing exosome-depleted FCS. Then, the exosomes were extracted from the supernatant using ExoQuick-TC. CD63 and calnexin levels were measured by Western blotting. Typical data of 3 independent experiments was shown. Other 2 data were shown in the **Supplementary Figure 2A**. **(B)** Electron microscope analysis of the exosomes obtained from HEK293T cells. Scale bar: 1 μM. **(C)** HEK293T cells were transfected with Flag-IRE1α construct and cultured in DMEM containing exosome-depleted FCS for 24 h. IRE1α overexpression in the cell was confirmed by Western blotting. **(D)** The exosomes were extracted from the supernatant, and the mRNA levels of total and spliced forms of XBP1 (sXBP1) were analyzed in cells and exosomes by RT-PCR, with specific primers for XBP1 (uXBP1, unspliced XBP1; sXBP1, spliced XBP1). Typical data of 5 independent experiments was shown. Other 4 data were shown in the **Supplementary Figure 2B**. ^∗∗^*P* < 0.01; ^∗^*P* < 0.05 by Bonferroni–Dunn test.

Results are expressed as the mean ± SEM. Statistical analyses were performed using Student’s *t*-test (for **Figure 1C**) or Bonferroni-Dunn test (for **Figure 2D**).

## Results

### sXBP1 mRNA in Cells Was Incorporated Into the Exosomes

We first collected the exosomes from MIN6 pancreatic β cells and measured the levels of exosome marker of pancreatic cells, CD81 (Guay et al., 2015). In addition to whole cell lysates of cells, we observed the expression of CD81 in the exosomes of MIN6 pancreatic β cells (**Figure 1A**). We did not detect calnexin in MIN6 pancreatic β cells exosomes, which is known to be expressed only in the cells (**Figure 1A**). Furthermore, we detected exosome by electron microscope analysis (**Figure 1B**). These results suggest that the exosomes were efficiently collected and the extract of the exosome may not contaminate detectable levels of cellular debris. The sXBP1 transcript is expressed in high levels in pancreatic cells (Iwawaki et al., 2004), and sXBP1 is constitutively expressed in pancreatic β cells (Engin et al., 2014). We observed the expression of mRNAs belonging to sXBP1 and an unspliced form of XBP1 (uXBP1) in MIN6 pancreatic β cells as assed by RT-PCR (**Figure 1C**, left band of the panel). We then checked whether XBP1 mRNA in the cells was incorporated into the exosomes of MIN6 pancreatic β cells. We performed RT-PCR analysis with specific primers for XBP1, and found that sXBP1 as well as uXBP1 were expressed in the exosomes (**Figure 1C**, right band of the panel). The levels of spliced/total XBP1 mRNA in the exosomes was higher than that of cells (**Figure 1C**). This suggests that sXBP1 mRNA was incorporated into the exosomes.

### sXBP1 mRNA in the Cells Was Incorporated Into the Exosome of HEK293T Cell, Which Overexpress IRE1α

We collected the exosomes from the cellular supernatant and analyzed the expression of exosome marker, CD63. We observed CD63 expression in whole cell lysates of cells and in the exosomes of HEK293T cells (**Figure 2A**). We did not detect calnexin in the exosomes of HEK293T cells, which is known to be expressed only in the cells (**Figure 2A**). Thus, the result indicates no contamination of cellular compartment in the exosome. Furthermore, we detected exosome by electron microscope analysis (**Figure 2B**). These results suggest that the exosomes were efficiently collected and the extract of the exosome may not contaminate detectable levels of cellular debris. We then checked whether XBP1 mRNA in the cells was incorporated into the exosomes of IRE1α overexpressed cells, as IRE1α overexpression increases XBP1 splicing through its autophosphorylation (Tirasophon et al., 2000). IRE1α construct was transfected into HEK293T cells and overexpression of IRE1α in the cells was confirmed by Western blotting (**Figure 2C**). We also confirmed the increase of sXBP1 mRNA levels in the cells at IRE1α overexpressed HEK293T cells by RT-PCR analysis (**Figure 2D**, left panel). We then analyzed the mRNA levels of uXBP1 and sXBP1 in the exosomes of these cells. As shown in **Figure 2D**, we observed higher levels of spliced/total XBP1 mRNA in the exosomes of HEK293T cells, both in mock- and IRE1α-transfected cells, than those in cells (**Figure 2D**, right panel). These results suggest that sXBP1 mRNA in the cells was incorporated into the exosomes. In addition, measurement of the levels of uXBP1 and sXBP1 indicated that the sXBP1 mRNA may have been incorporated in the exosomes of HEK293T cells (**Figure 2D**).

## Discussion

sXBP1 acts as a transcription factor, regulating cellular homeostasis by coping with stress (Yoshida et al., 2001). In the present study, we found that XBP1 and sXBP1 mRNAs were both expressed in the exosomes. We also found that the levels of spliced/total XBP1 mRNA in the exosomes were higher when compared with its levels inside the cells. To date, the mechanisms involved in the incorporation of sXBP1 mRNA into the exosomes remain unelucidated. It has been reported that the formation of multivesicular bodies (MVBs) increases by ER stress (Kanemoto et al., 2016). Because the production of MVBs and their fusion with the plasma membranes (PM) plays a key role in the release of exosomes (van Niel et al., 2006), incorporation of sXBP1 mRNA would likely occur during this process. Currently, we do not know the molecular mechanisms of the incorporation of sXBP1 mRNA in the exosomes, which would be interesting future subjects. IRE1α was suggested to play a role in releasing exosomes by ER stress (Kanemoto et al., 2016). Therefore, it would also be an interesting subject to analyze the molecular mechanisms how IRE1α-XBP1 pathway was involved in releasing exosomes as well as incorporating sXBP1 mRNA in the exosome.

sXBP1 transcripts are highly expressed in pancreatic cells (Iwawaki et al., 2004) (**Figure 1**). In addition, exosomes can cross the blood–brain barrier (Alvarez-Erviti et al., 2011; Chen et al., 2016), a fact that could be exploited in treating neurodegenerative diseases (Jarmalavièiūtë and Pivoriûnas, 2016). Epidemiological evidence suggests that type 2 diabetes is a risk factor in the development of AD (Leibson et al., 1997; Pasinetti and Eberstein, 2008), and pancreatic β cell function may be closely linked to AD onset. If pancreatic exosomes are absorbed by neurons, then they could be key to regulating neuronal function. sXBP1 was suggested to be involved in protecting amyloid β-induced neurotoxicity in Alzheimer’s disease (AD) models (Casas-Tinto et al., 2011). Therefore, it would be an interesting future topic to investigate possible involvement of sXBP1 in the exosome in AD progression.

The manner in which ER stress signals are transmitted extracellularly remains unexplored. We found that one of the ER stress signal-induced transcripts, sXBP1, was incorporated into the exosomes. Our results suggest that exosomes may play a vital role in the extracellular release of ER stress signals. To our knowledge, this is the first report on incorporation of ER stress signal in the exosomes, and our results provide novel insights into the exosomal transmission of intercellular signal.

## Author Contributions

MN performed the experiment. TH and MN analyzed the data. TH conceived hypothesis, interpreted the data, and wrote the manuscript. KO discussed and checked the manuscript.

## Conflict of Interest Statement

The authors declare that the research was conducted in the absence of any commercial or financial relationships that could be construed as a potential conflict of interest.
