# Supplementary figures and images for: Incorporation of the Endoplasmic Reticulum Stress-Induced Spliced Form of XBP1 mRNA in the Exosomes
Source: Front Physiol. 2018 Sep 26;9:1357. doi: 10.3389/fphys.2018.01357 (PMC6168632; doi:10.3389/fphys.2018.01357)

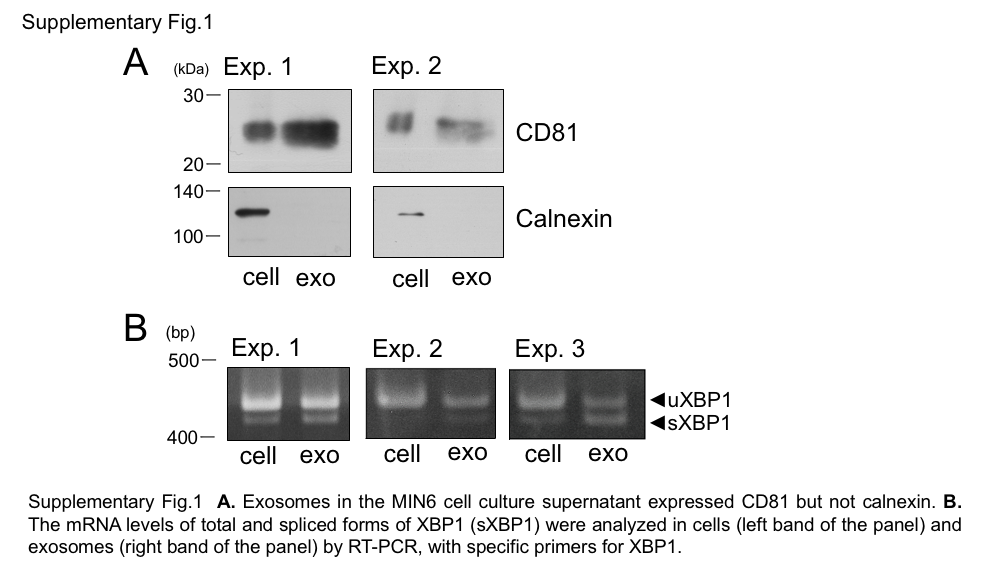

Supplement: Supplementary file 1 [file Image_1.tiff]

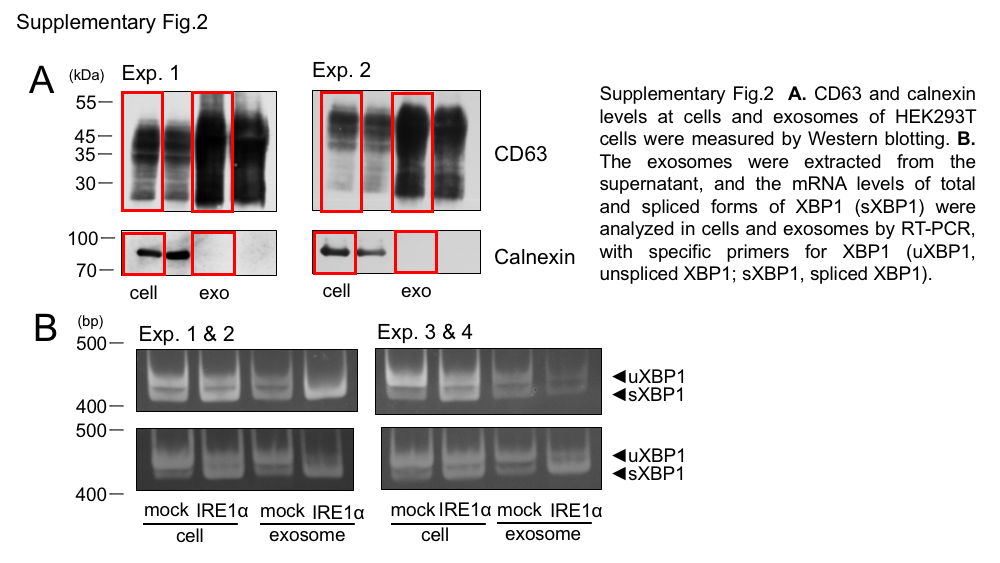

Supplement: Supplementary file 2 [file Image_2.tiff]
